# Balance, Gait Kinematics, and Fear of Falling After a Four-Month Targeted Training Program in a Patient with Cervical Dystonia: A Case Report

**DOI:** 10.3390/ijerph22121831

**Published:** 2025-12-06

**Authors:** Oscar Crisafulli, Marta Sarrocco, Matteo Fortunati, Marco Serra, Venere Quintiero, Giuseppe D’Antona

**Affiliations:** 1 Centro di Ricerca Interdipartimentale Attività Motorie e Sportive (CRIAMS)-Sport Medicine Centre Voghera, University of Pavia, 27058 Voghera, Italy; oscar.crisafulli@unipv.it (O.C.); marta.sarrocco01@universitadipavia.it (M.S.); matteo.fortunati@students.uniroma2.eu (M.F.); serramarco.noli@gmail.com (M.S.); venere.quintiero01@universitadipavia.it (V.Q.); 2 Department of Industrial Engineering, University of Tor Vergata, 00133 Rome, Italy; 3 Department of Public Health, Experimental and Forensic Medicine, University of Pavia, 27100 Pavia, Italy

**Keywords:** cervical dystonia, exercise therapy, rehabilitation, balance, gait

## Abstract

In cervical dystonia (CD), balance and gait impairments can compromise daily activities and negatively affect quality of life. However, interventions addressing these deficits remain poorly investigated. A 54-year-old woman with CD, presenting balance and gait difficulties that interfered with work-related motor tasks, underwent a four-month training program. Sessions (40 min, three times per week) combined lower-limb strengthening, proprioceptive and balance exercises, and integrated motor–cognitive tasks. Pre- and post-intervention assessments included gait speed (GS), stride length (SL), and stance time (ST) under usual (UW), fast (FW), and dual-task (DT) walking conditions, measured with an inertial sensor (BTS G-Walk). DT cost was calculated for GS and SL. Balance was evaluated with the Mini-BEST and Four-Square Step Test (FSST), while fear of falling was measured with the Falls Efficacy Scale-International (FES-I). Of note, both assessment sessions were conducted in the absence of botulinum toxin effects, whereas the training was performed, at least in part, under its influence. After training, increase were observed in GS and SL, with reductions in ST across all gait conditions. DT cost decreased for both GS and SL. Balance performance increased, and fear of falling was reduced. Importantly, the patient reported a marked improvement in work-related performance. This case suggests that a specific training program may effectively ameliorate balance and gait in CD, with positive effects on functional mobility. Further studies on larger samples are warranted to confirm efficacy.

## 1. Introduction

Cervical dystonia (CD) is a rare neurological disorder [1] characterized by involuntary contractions of the neck muscles, resulting in abnormal head and neck postures [2]. The pathophysiology of CD is multifaceted, with growing evidence suggesting that the disorder arises from dysfunction within neural circuits involving the basal ganglia, cerebellum, thalamus, and cortical regions [3,4]. Limitations in the range of motion and pain constitute the most significant clinical consequences and, together with psychiatric symptoms, may result in a substantial degree of disability [5]. Moreover, several studies have documented impairments in balance and gait functions [6,7,8]. The available data suggest that balance impairments could mainly arise from disrupted sensorimotor integration of visual, vestibular and proprioceptive inputs [6,9,10], which constitutes a fundamental neurophysiological component underlying postural control [11,12,13].

The first-line treatment is intramuscular injections of botulinum toxin (BoNT), which are effective in reducing muscle rigidity, pain, and involuntary contractions, as well as improving head posture [14]. However, these injections must be repeated every three to four months as their effects gradually wear off and the muscle returns to its baseline condition [15]. Additionally, physical therapy can be useful in reducing pain [16], decreasing overactivity in the affected neck muscles, and enhancing range of motion [17]. However, to date, specific interventions aimed at improving balance and gait in these patients remain underexplored. The issue is relevant since, as observed in various at-risk populations such as older adults and patients with Parkinson’s disease, gait and balance are major determinants of fundamental domains, including daily life autonomy [18,19], physical activity level [20], and quality of life [21]. Importantly, their impairment also exposes individuals to an increased risk of falls [22]. Few studies have examined these aspects in CD, but the available data points out several critical issues. For instance, a cross-sectional study involving 46 CD patients documented both an increased risk of falls and a heightened fear of falling, which may contribute to reduced activity levels [23]. Similarly, a survey of 65 patients found that nearly half (45.8%) had experienced at least one fall in the preceding six months [24]. Overall, these data suggest that balance and gait impairments in CD can significantly impact independence and daily functioning, highlighting the need to identify effective strategies to address these deficits.

Data from other neurological conditions, such as Parkinson’s disease and multiple sclerosis, indicate that targeted physical training can significantly improve balance and gait functions in patients [25,26], with ameliorations on independence, fear of falling and overall quality of life [27,28,29]. However, this approach has never been tested in patients with CD.

Hence, this case report presents the pre- and post-intervention values for balance, gait and fear of falling following a 4-month targeted training program in a patient with CD, aiming to provide preliminary insights that may inform future research on rehabilitation approaches and clinical management of mobility deficits in these patients.

## 2. Case Presentation

**Figure 1:**
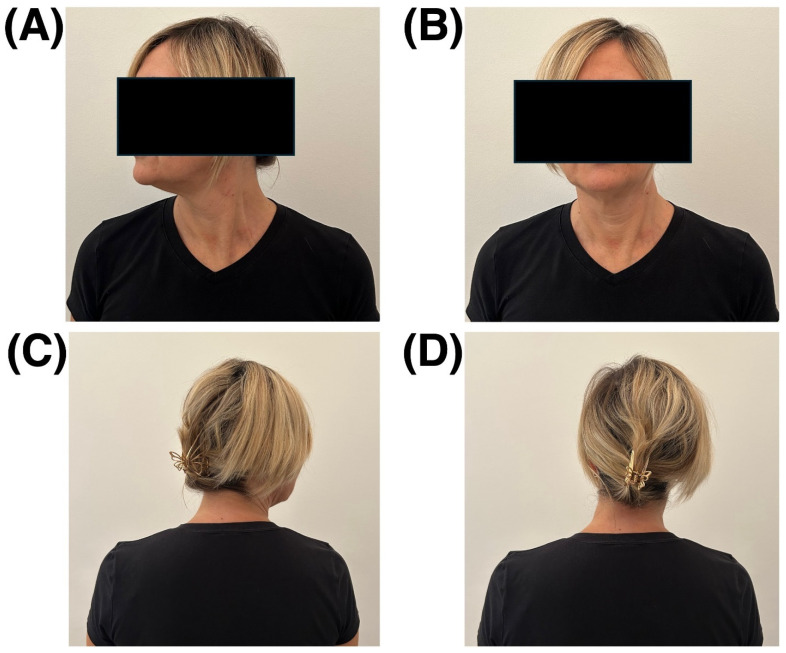
Frontal-plane photographs of the patient’s neck and head: anterior view (**A**,**B**) and posterior view (**C**,**D**). In panels (**A**,**C**), the patient was instructed to keep the muscles relaxed and assume her natural posture. In panels (**B**,**D**), she maintained her habitual postural correction attempt. Written permission to publish the photos was obtained from the patient.

The patient was a 54-year-old female affected by CD, with no comorbidities. She had been diagnosed in 2007 at the age of 37. In July 2011, she received her first BoNT injection and has continued treatment approximately every 3–4 months since then, reporting significant benefits in terms of pain relief and range of motion. In September 2024, she presented to our center reporting balance deficits that negatively affected her gait and work-related activities. For instance, her job in a supermarket frequently required tasks she found challenging, such as walking at a sustained pace, climbing small staircases, and reaching or arranging items on elevated shelves. Of note, despite such difficulties, no episodes of falling were reported; however, alarming near-fall events occurred several times and were a major reason motivating the patient to seek preventive interventions. Her body weight was 70 kg and her height was 1.70 m. At the neurological evaluation, normal strength (both segmental and global), muscle trophism, and tone (proximal and distal) were observed. The main postural alteration of the head was a marked rotation towards the right of approximately 70°, which was appreciable only when the patient was explicitly asked to relax her neck muscles. This was because she habitually maintained an almost constant correction of her head position. In addition, she exhibited moderate right laterocollis and mild right shoulder elevation (Figure 1). Of note, the patient reported that her last BoNT injection had been administered at least four months prior to the examination; therefore, the observations reported are unlikely to have been influenced by its effects [15].

For the last BoNT injection prior to referral to our clinic, the patient received intramuscular injections targeting multiple cervical muscles. On the right side, 75 units were administered to the occipitalis capitis, 150 units to the splenius capitis divided across two sites, and 100 units to the longissimus capitis. On the left side, 75 units were injected into the sternocleidomastoid and 50 units into the semispinalis capitis. The choice of injection sites appears consistent with the postural alterations observable in the absence of BoNT effect. On the Toronto Western Spasmodic Torticollis Rating Scale (TWSTRS) [30], she obtained a total score of 33, including 10 points on Part II, which evaluates disability. The Fahn-Tolosa-Marin Rating Scale [31] revealed no evidence of tremor. The patient was enrolled in a training program aimed at improving balance and gait, with domain-specific assessments conducted before and after the intervention, alongside fear of falling evaluations.

## 3. Evaluation Methods and Training Program

**Figure 2:**
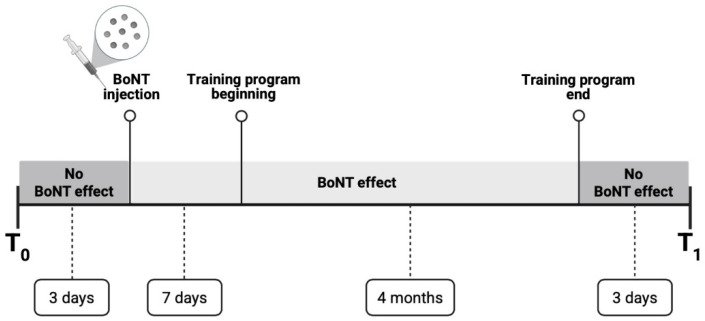
Representative timeline of events. The baseline evaluation (T0) was performed approximately 4 months after the last BoNT injection, and was therefore presumably unaffected by the toxin. Three days after this assessment, the patient underwent a new BoNT injection, and one week later she initiated the 4-month training program. Since the duration of the training program roughly corresponded to the expected efficacy period of BoNT, it is assumed that the training was performed under its influence, whereas the post-training evaluation (T1) was again conducted in the absence of its effects.

As mentioned, at baseline assessment (T0), approximately four months had elapsed since the patient’s last BoNT injection, thus excluding its effect [15]. The patient subsequently received a new injection between T0 and the start of the training program, which began seven days later. This injection was administered at the same sites and dosages as the last one performed before the patient came to our center. Consequently, the training was performed at least in part during the period of BoNT activity. The post-intervention assessment (T1) was carried out in the absence of the toxin’s effect, as the training program lasted four months [15]. BoNT can only slightly modify gait kinematics during its active period [32]. Hence, conducting both assessments during periods when BoNT is not expected to be active prevents the risk of potential bias. The temporal sequence of events is illustrated in Figure 2.

Importantly, the assessments at T0 and T1 were performed by two different evaluators (MF and VQ, respectively), both blinded to the patient’s training status. Using independent, blinded assessors reduces the risk of expectation bias and strengthens the accuracy of the outcome evaluation.

### 3.1. Gait Kinematics Assessment

Gait kinematics was assessed under three conditions: usual walking (UW), in which the patient was asked to walk at her self-selected comfortable speed; fast walking (FW), in which she was asked to walk at her maximum speed, without running; cognitive dual task (DT), in which she walked while performing a verbal fluency task. Specifically, the verbal fluency task required to walk while continuously producing words starting with a letter provided by the operator immediately before the trial. This task has previously been shown to be challenging for patients with CD [33]. The FW condition was evaluated because the patient described it as particularly challenging, whereas the DT condition was evaluated as a representative ecological walking paradigm [34]. Importantly, both conditions may constitute challenges for patients with CD [33,35]. In each condition, the patient walked back and forth along a 20 m path for 1 min, with a 5 min rest period between tasks. Each gait task was performed three times, with the order randomized. Gait parameters were measured using a wearable inertial device (G-Walk, BTS Bioengineering, Italy). The device consists of a triaxial accelerometer (16-bit/axis) with a sensitivity of ±2 g, a triaxial gyroscope (16-bit/axis) with a sensitivity of ±2000°/s, and a triaxial magnetometer (13-bit, ±1.200 µT). According to the manufacturer’s instructions, the sensor was positioned just below the line connecting the two dimples of the lumbosacral region (corresponding to S1–S2 vertebrae) using an elastic band. All data were acquired at 100 Hz and transmitted via Bluetooth to a notebook, then processed using BTS G-Studio software, version 3.5.25 (BTS Bioengineering, Milano, Italy). The software provided a set of gait parameters, from which the following were analyzed: gait speed (GS, m/s), stance time (ST, s), and stride length (SL, cm). Of note, the GS parameter included the time required to change gait direction. Results are expressed as the mean ± standard deviation (SD) of the three trials per walking condition. In line with previous reports [36,37], DT cost was calculated to better characterize performance during usual and DT walking. It was defined as ([dual-task − single-task]/single-task) × 100 and computed for two key gait parameters, GS and SL [38], based on the mean of the three trials for each of the two walking conditions.

### 3.2. Balance, Dynamic Stability and Fear of Falling Assessment

Balance and dynamic stability were assessed using the Mini-Balance Evaluation System Test (Mini-BEST) [39] and the Four-Square Step Test (FSST) [40], respectively. Fear of falling was assessed using the Falls Efficacy Scale International (FES-I) [41]. All these tests have already been employed in patients with CD [23,33].

### 3.3. Training Program

At her first visit to our center, the patient reported engaging in physiotherapist-prescribed exercise for approximately five years, performing sessions twice weekly. Each session lasted 30–35 min, comprising 20 min of stationary cycling followed by 10–15 min of general stretching, with particular focus on the dystonic muscles. Pre-study exercise patterns were reported to be stable over this period. Our program was integrated to this ongoing routine.

The proposed training consisted of three sessions per week on non-consecutive days, alternating with the patient’s two usual exercise sessions and rest over the weekend. Each session lasted approximately 40 min, comprising a 5 min warm-up, a central phase of about 30 min, and a 5 min cool-down. The warm-up included joint mobility exercises for both upper and lower limbs (e.g., trunk rotations and tilts), followed by walking drills (toe walking, heel walking, and heel-to-toe gait), while the cool-down involved stretching exercises for the lower limbs. These phases were consistent across all sessions, whereas the central training phase varied, as described below. In the absence of the literature on balance and gait training in CD, the choice of exercises was based on those previously shown to be effective in other cohorts with balance and gait impairments, such as patients with other neurological or neuromuscular disorders and older adults. Each training session was supervised by qualified personnel, which not only ensured patient safety during exercise execution, but also allowed the exercises to be carefully adapted to the patient’s capabilities. Additionally, supervision also enabled monitoring of adherence to the program and any potential adverse events, none of which occurred.

In the first session of the week, the patient performed bodyweight resistance training exercises for the lower limbs, including squats, sagittal and frontal plane lunges, and single-leg step-ups on an elevated surface. Squats and lunges were performed on unstable surfaces (Skimmy, Navaris), as evidence suggests that this modality is more effective for improving dynamic balance compared to training on stable surfaces [42,43]. Furthermore, given that the tibialis anterior, soleus, and peroneal muscles play a crucial role in balance control [44,45], specific strengthening exercises for these muscles using elastic bands were incorporated. These exercises were included because lower limb strength is a key contributor to both balance and gait performance, as demonstrated in various cohorts [18,46,47].

The second session focused on balance and proprioception training, including complex tasks such as maintaining single-leg balance while reaching for objects on the ground, touching objects positioned around the patient, or performing the tasks on an unstable surface. Additionally, bilateral balance exercises were carried out on unstable surfaces, with progressive challenges such as performing the tasks with eyes closed or bouncing a ball against a wall. These exercises have been shown to improve postural control in various cohorts of individuals with balance impairments [48,49,50].

The third training session integrated elements from the first and second sessions. Resistance exercises were administered according to the same protocol as in the first session, whereas proprioceptive and balance tasks were performed with the addition of a concurrent cognitive load (verbal fluency or backward counting). The motor–cognitive training approach has been reported to improve both single- and dual-task balance and gait in neurological populations [51,52,53].

Exercise intensity was determined based on the patient’s self-assessment using the Borg Category Ratio (CR) 0–10 scale [54], which has already been employed for exercise prescription in various pathological contexts [55,56,57]. Specifically, she was instructed to maintain an effort corresponding to a Borg CR of 5–7, indicative of the “heavy” category. When an exercise became too easy, it was adjusted in terms of sets, repetitions, or (for balance and proprioceptive exercises) duration, to remain within the prescribed range. Based on patient feedback, recovery time was set at approximately 2 min for resistance exercises and 1 min for balance exercises.

Notably, regarding the duration of the training period, session frequency, total number of sessions, duration of individual sessions, and overall weekly training volume, the proposed protocol aligned with the quantified dose–response relationships of balance training in healthy older adults [58], which served as a reference in the absence of specific guidelines for CD. Table 1 summarizes the training details, including sets, repetitions/time per exercise, and the progression of workload from the initial to the final week of the program.

## 4. Results and Discussion

**Figure 3:**
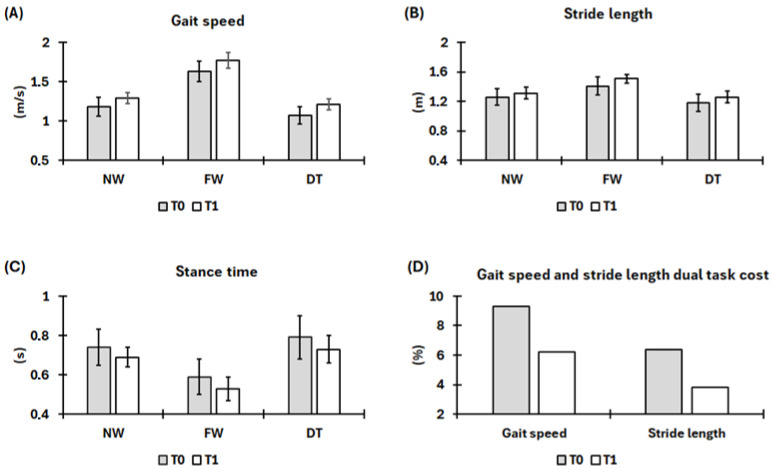
Mean values of (**A**) gait speed, (**B**) stride length, and (**C**) stance time under usual (UW), fast (FW), and dual-task (DT) walking conditions. Panel (**D**) shows the dual-task cost for gait speed and stride length. Gray columns represent baseline values (T0), while white columns represent post-training values (T1). Black bars indicate standard deviation (SD).

In this study, we preliminary evaluated the potential impact of a 4-month training program aimed at improving balance and gait on functional outcomes and fear of falling into a patient with CD. The results would point out improvements across all the domains investigated. Regarding gait kinematics, GS increased under all three tested conditions (UW: from 1.18 ± 0.12 m/s to 1.29 ± 0.07 m/s; FW: from 1.63 ± 0.13 m/s to 1.77 ± 0.10 m/s; DT: from 1.07 ± 0.11 m/s to 1.21 ± 0.07 m/s), a result indicative of better efficiency in locomotion and enhanced mobility [59,60,61]. An additional indication of enhanced gait ability is given by SL [62], which increased across all the conditions tested (UW: from 1.26 ± 0.11 m to 1.31 ± 0.08 m; FW: from 1.41 ± 0.12 m to 1.51 ± 0.06 m; DT: from 1.18 ± 0.12 m to 1.26 ± 0.08 m). Finally, ST decreased under all gait conditions (UW: from 0.74 ± 0.09 s to 0.69 ± 0.05 s; FW: from 0.59 ± 0.09 s to 0.53 ± 0.06 s; DT: from 0.79 ± 0.11 s to 0.73 ± 0.07 s), suggesting improvements in postural control, confidence during single-leg support, and gait dynamics [63,64]. In addition, the DT cost decreased for both GS (from 9.32% to 6.20%) and SL (from 6.35% to 3.82%), a finding that would indicate a more efficient allocation of cognitive resources and a reduced attentional demand during the motor task [52]. Moreover, the consistent reduction in SD suggests minor gait variability, improved neuromotor control and increased safety during gait [65,66]. Figure 3 presents a graphical summary of the results.

Overall, the observed improvements may be attributed to the proposed training program, as similar protocols have yielded comparable outcomes in other neurological populations [67,68], including enhanced DT performance [52]; however, it should be noted that the study design does not permit conclusions regarding causality.

Interestingly, baseline values appear in line with those observed in a cohort of 17 CD patients, in whom gait kinematics were assessed under the same conditions as those applied to our patient (UW, FW and DT) [33]. In this regard, the increases in GS and the reductions in ST under FW and DT conditions appear particularly relevant, as the significant differences relative to healthy controls reported by Crisafulli et al. (2021) [33] indicate such parameters as particularly critical for CD patients.

With respect to the UW condition, contextualization of the T0 data against the mean values from a cohort of 94 healthy women of similar age and anthropometric characteristics [69] indicates that the observed values are lower for GS (1.18 ± 0.12 m/s vs. 1.35 ± 0.17 m/s) and SL (1.26 ± 0.11 m vs. 1.40 ± 0.12 m), and higher for ST (0.74 ± 0.09 s vs. 0.66 ± 0.06 s). Interestingly, at T1, although the trend persisted, the values showed a clear shift toward the aforementioned normative values (GS: 1.29 ± 0.07 m/s vs. 1.35 ± 0.17 m/s; SL: 1.31 ± 0.08 m vs. 1.40 ± 0.12 m; ST: 0.69 ± 0.05 s vs. 0.66 ± 0.06 s), suggesting that the proposed intervention improved the patient’s functional performance. Noteworthy, the increase in GS meets the threshold for minimal clinically important differences for change in comfortable GS of adults with pathology [70], further supporting the training efficacy. Importantly, given the single-subject design, it is not possible to calculate a precise minimal detectable change (MDC) for gait in this patient; however, the magnitude of observed changes suggests they are likely meaningful and exceed typical measurement variability reported in other adult populations tested with the same device [71].

Improvements in balance were also observed, as evidenced by an increase in the mini-BEST test score (from 19 to 28 points) and a decrease in the time taken to complete the FSST (from 6.92 s to 5.84 s) (Table 2). Again, these improvements may have been induced by the training program, based on results obtained from similar protocols in elderly and neurological patients [72,73]. Notably, the simultaneous improvements in balance and gait may be, at some extent, interrelated since their association has been previously documented in patients with CD [33], as well as in others neurological cohorts [48,74,75]. Of note, improvements in these domains may have contributed to a reduction in the level of disability. In fact, at T1, the TWSTRS total score decreased by 4 points, driven by reductions in Section II (Disability Scale) (Table 2), with the “Work” and “Activities Outside Home” subsections decreasing from 2 to 0 points. This finding is consistent with the patient’s report of marked improvements in work capacity and in many daily motor activities, which progressively increased as the program advanced. The patient reports that these perceived improvements motivated her to diligently follow the proposed plan, which has now become an integral part of her exercise routine. It should be noted that the motivation shown by the patient, as well as the fact that the activity was supervised [76], may have contributed to the observed improvements.

Finally, patients’ improvement in balance may explain the reduction in fear of falling, suggested by the 9-point decrease in the FES-I score after the intervention (Table 2). This seems coherent with previous evidence from elderly populations [77] and individuals with Parkinson’s disease [29], where balance-oriented training has been associated with lower FES-I scores. Results before and after the intervention regarding balance, dynamic stability, fear of falling and TWSTRS assessment are summarized in Table 2.

### Limitations and Future Directions

The results, being based on a single patient, have no inferential value and should be interpreted only as indicative of the potential for testing the same intervention in a larger patient cohort. Inclusion of an adequately sized cohort, a CD patient control group, and protocol randomization will be essential for establishing causal relationships between the intervention and the observed outcomes. Additionally, although fear of falling was one of the main reasons that led the patient to seek our intervention, she had never actually experienced a fall, only several near-fall events. Future studies should evaluate whether this type of intervention has different effects on balance, gait, and fear of falling in a group of patients who have experienced falls compared to a group, like in the present case, who have not, and whether it can reduce the number of falls in the former group. Additionally, potential learning effects related to repeated gait and balance assessments, regression to the mean, natural fluctuations of CD symptoms and the placebo-like effect of intensive supervised training, cannot be excluded and may have influenced the reported results to some extent. Moreover, it is currently unknown whether training performed with or without the influence of BoNT yields similar results. Clarifying these aspects would be important to better understand the potential gains achievable through specific balance and gait rehabilitation in these patients and to provide improved context for the proposed interventions. A follow-up period will be crucial to assess the long-term retention of the results in these patients. Finally, only a single baseline measurement (T0) was performed. The lack of repeated baselines is explicitly recognized as a limitation of this case report.

## 5. Conclusions

Following the proposed balance and gait training, performed, at least in part, under BoNT effect, this patient reported improvements in functional performance and fear of falling. These results suggest the potential utility of the proposed intervention and encourage the possibility of replicating the approach in a larger patient cohort to confirm efficacy.

## Figures and Tables

**Table 1:** Training Sessions Plan and Progression.

**First Session: Resistance Training**		
	**Initial week**	**Final week**
*Exercises*	*Sets per repetition*	*Sets per repetition*
Unstable body-weight squat	3 × 8	3 × 10
Unstable body-weight lunges		
Forward lunges	2 × 8, each leg	3 × 10, each leg
Backward lunges	2 × 8, each leg	3 × 10, each leg
Lateral right lunges	2 × 8, right leg	3 × 10, right leg
Lateral left lunges	2 × 8, left leg	3 × 10, left leg
Single-leg step-ups	2 × 8, each leg	3 × 10, each leg
Elastic bands strengthening exercises for tibialis anterior, soleus, and peroneal muscles	3 × 8 each exercise to perform as a circuit	4 × 10 each exercise to perform as a circuit
**Second session: balance and proprioception training**		
*Exercises*	*Sets per repetition or time duration*	*Sets per repetition or time duration*
Single-leg balance with object reaching	4 × 15 s, each leg	5 × 30 s, each leg
Unstable single-leg balance	3 × 10 s of position maintenance, each leg	4 × 15 s of position maintenance, each leg
Unstable bilateral balance exercise	4 × 10 s of position maintenance	5 × 15 s of position maintenance
Unstable bilateral balance with eyes closed	3 × 10 s of position maintenance	4 × 15 s of position maintenance
Unstable bilateral balance while bouncing a ball against a wall	3 × 10 of bouncing a ball	4 × 15 of bouncing a ball
**Third session: motor–cognitive training**		
*Exercises*	*Sets per repetition or time duration*	*Sets per repetition or time duration*
Unstable body-weight squat	2 × 8	2 × 10
Unstable body-weight lunges		
Forward lunges	1 × 8, each leg	2 × 10, each leg
Backward lunges	1 × 8, each leg	2 × 10, each leg
Lateral right lunges	1 × 8, right leg	1 × 10, right leg
Lateral left lunges	1 × 8, left leg	1 × 10, left leg
Single-leg step-ups	2 × 8, each leg	2 × 10, each leg
Single-leg balance with object reaching *	2 × 20 s, each leg	3 × 30 s, each leg
Unstable single-leg balance *	2 × 10 s of position maintenance, each leg	2 × 20 s of position maintenance, each leg
Unstable bilateral balance exercise *	2 × 10 s of position maintenance	3 × 10 s of position maintenance
Unstable bilateral balance with eyes closed *	1 × 10 s of position maintenance	2 × 15 s of position maintenance
Unstable bilateral balance while bouncing a ball against a wall *	1 × 10 of bouncing a ball	2 × 15 of bouncing a ball

* Exercise performed with a concurrent cognitive task.

**Table 2:** Pre- and post-training balance-related and TWSTRS assessments.

Balance Related and TWSTRS Assessments	Pre-Training Values	Post-Training Values	Absolute Change
Mini-BEST (score)	19	28	+9
FSST (s)	6.92	5.84	−1.08
FES-I (score)	30	21	−9
TWSTRS total (score)	33	29	−4
TWSTRS part 2 (score)	10	6	−4

Mini-BEST, Mini-Balance Evaluation System Test; FSST, Four-Square Step Test; s, seconds; FES-I, Falls Efficacy Scale International; TWSTRS, Toronto Western Spasmodic Torticollis Rating Scale.

## Data Availability

The original contributions presented in the study are included in the article, further inquiries can be directed to the corresponding author.
